# Primum non nocere: a critical analysis of early intervention clinical trials in smoldering multiple myeloma

**DOI:** 10.3389/fonc.2026.1834686

**Published:** 2026-09-22

**Authors:** Harsh Parmar, Noa Biran, Pooja Phull, Kimberly Doucette, James Di Palma-Grissi, Joseph Franz, David Vesole, David Siegel

**Affiliations:** Division of Multiple Myeloma, Department of Oncology, John Theurer Cancer Center at Hackensack University Medical Center, Hackensack, NJ, United States

**Keywords:** asymptomatic myeloma, clinical trials, interventional clinical trials, multiple myeloma, smoldering myeloma (SMM)

## Abstract

The approach to the management of smoldering multiple myeloma has changed over the years, which has evolved from observation towards early therapeutic intervention. Yet, a comprehensive examination of several key clinical trials reveals methodological challenges and limitations, ranging from inconsistent risk stratification of SMM to biased interpretations of results due to artifactual inflation of benefit arising from lead-time bias. Intervention for patients with a largely asymptomatic disease needs to be re-assessed carefully, where toxicities arising from treatment are not well justified. Until a more precise risk stratification is developed, the principle of “primum non nocere” (first, do no harm) should curb enthusiasm for early intervention in a condition in which a substantial number of patients will remain progression-free without treatment.

## Introduction

Since its recognition as a clinically distinct entity in 1980 (1, 2), there has been considerable evolution in the approach towards management of smoldering multiple myeloma (SMM) (3–7). The traditional watchful monitoring method gained recognition as a standard paradigm considering two key concepts- 1. Avoid unnecessary exposure to therapy in patients with a potentially indolent disease and 2. Preserve disease sensitivity to therapies when patients need it most. Principally, this paradigm has been challenged several times by the idea that early treatment would prevent end-organ compromise and may eradicate it completely, leading to a cure at a time point when the disease burden is low and therefore easier to treat (8, 9). In 2014, the historic “CRAB” criteria (Hypercalcemia, renal failure, anemia and bone disease) were expanded to “SLiM CRAB” (bone marrow plasmacytosis >=60%, serum free light chain (FLC) ratio>=100, more than one MRI-defined focal bone lesion>0.5mm) to further re-classify patients into the active myeloma category (10). This expansion was based on the initial assumption that approximately 70-80% of ultra-high-risk patients would eventually progress to active disease within two years (11, 12), an assumption that subsequently proved to be an overestimation of the actual risk (13). Therapeutic interventions were subsequently implemented in the smoldering phase of the disease with the primary aim of reducing the morbidity risks associated with progression, particularly those related to skeletal or renal events (14–17).

Recent clinical trials in SMM represent a departure from the traditional myeloma research approaches and practices. To understand the rationale for early therapeutic intervention in SMM, it is essential to grasp the concept of SMM risk stratification. Several risk models have been proposed historically but they continue to show inconsistencies when compared to each other (18). As has been described multiple times in the past, SMM, rather than being a disease entity requiring early intervention, is simply a statistical concept that provides a numerical prediction for developing multiple myeloma (19). Such a notion suggests a more fluid continuum of disease progression rather than discrete stages, which necessitates a re-evaluation of our current algorithms and risk assessment methodologies.

## Risk stratification models

One of the very first retrospective studies was conducted by the PETHEMA group, which investigated the risk of progression in patients with monoclonal gammopathies, including multiple myeloma (MM) and SMM, using multiparametric flow cytometric analysis of bone marrow aspirate samples (20). Patients harboring >=95% aberrant plasma cells in the bone marrow compartment at diagnosis had a higher risk of progression. While the study led to the development of distinct categories for both MGUS and SMM, fundamentally changing our approach to risk stratification of these conditions and providing essential insights into risk assessment using MFC, it did not address a significant concern about the reproducibility of results when using the technique across laboratories. Additionally, the small sample size and retrospective design of the study limited the generalizability of these findings.

In 2008, Mayo Clinic proposed another risk model based on three variables, including serum monoclonal protein level, bone marrow plasma cell percentage and serum free light chain ratio (21). Each variable was assigned a point if it exceeded a predetermined threshold. A cumulative score of the three risk-stratified patients into low, intermediate, and high-risk for progression categories. The model is easy to apply using simple laboratory parameters readily available to clinicians in routine practice, although the binary cutoffs, established using retrospectively collected data, introduce bias and are overly simplistic for a very complex condition such as SMM for risk prediction.

The PETHEMA and Mayo 2008 model were derived before the recognition of ‘myeloma defining events’ including those of a serum free light chain ratio of >100 and evidence of more than one intramedullary plasmacytoma >0.5cm as observed on an MRI both which now satisfy the criteria for active multiple myeloma, as a result of which, it is possible that these subgroups of patients were included in both models, which may have artifactually inflated the progression rates in the ‘high risk’ categories and therefore no longer applicable in the contemporary era (10).

In 2018, Mayo Clinic developed a newer risk stratification, popularly known as the ‘ 20-2-20 ’ model, that once again produced bifurcation of variables using thresholds established using retrospective data, which did, however, succeed the newer International Myeloma Working Group (IMWG) definition of multiple myeloma, making a more apparent distinction between active and symptomatic myeloma. One must, however, interpret this data cautiously as only 30% of patients underwent advanced imaging at diagnosis, which may have misclassified patients into ‘high-risk’ categories and confounded the results (22).

Using static risk parameters at diagnosis does not account for dynamic changes over time, which highlights the limitations of viewing SMM as a discrete entity rather than a continuum, a fallacy commonly shared by all three models. Indeed, the eligibility of the majority of patients in the interventional SMM trials was determined by a single laboratory evaluation. Moreover, as the authors recognize, referral patterns to a specialized tertiary care center create an inherent selection bias that may overrepresent complex cases. The methodologies adopted in all three models involved dichotomizing continuous variables using absolute thresholds, which often results in information loss. A more granular approach was adopted by the IMWG 2020 risk model, which proposed a 17-point scale with 4-level stratification for each variable, potentially overcoming the prior limitation of dichotomous variable classification in earlier models (23). Additionally, the risk of progression to active MM is not uniformly determined by all variables, and their relative contributions should be assessed using hazard ratios rather than treated as equivalent binary factors. Earlier models insufficiently addressed this issue by assuming that each variable carried an equal risk of progression once the established threshold was exceeded, without accounting for the non-uniform magnitude of their impact. The IMWG model also attempted to address this issue by assigning points based on the magnitude or impact of variables for progression, as determined by their respective hazard ratios. Unfortunately, due to the model’s complexity, it is rarely used in clinical practice. Moreover, validation of the IMWG model by other groups is still pending. Once again, the risk model utilizes static parameters determined at the time of diagnosis and fails to account for dynamic changes over time.

PANGEA (Precursor Asymptomatic Neoplasms by Group Effort Analysis) model offered a more promising approach, incorporating longitudinal serial biomarker assessments rather than isolated measurements, directly addressing one of the fundamental limitations of all prior models (24). It demonstrated superior predictive capability and enabled more precise risk stratification, representing a conceptual shift from viewing SMM as a discrete entity with fixed risk estimates and perhaps aligning best with our understanding of SMM. Despite this significant advancement, it has several constraints, including computational complexity, retrospective nature, lack of evidence of reproducibility (not yet validated), and the assumption of a linear relationship between biomarkers and progression (as defined by SLiM-CRAB). Therefore, its use in clinical practice has not been widely adopted.

A better stratification model would need to address two important deficiencies: the loss of prognostic information with binary classification and the inability to capture disease trajectory over time. A hybrid model incorporating the granular hazard ratio-based multi-point scale of the IMWG 2020 stratification with the longitudinal serial biomarker assessment framework of PANGEA would closely approximate the true biology of SMM as a disease continuum. Such a model would capture the prognostic magnitude of each variable while updating scores dynamically through serial measurements, addressing the static limitations shared by all models prior to PANGEA. A near-universal flaw shared by all models and outcome assessments across the entire specialty of oncology is the overreliance on the Kaplan-Meier estimate, which is methodologically familiar but, as a non-parametric estimate, becomes increasingly unreliable as the at-risk denominator decreases over time. Parametric survival models (e.g., Weibull, Royston-Parmar) would likely provide more statistically rigorous estimates of risk and treatment effects. Therefore, integrating dynamic biomarker data for risk stratification with a multi-point scale based on HR and a better assessment methodology using parametric estimates would represent an important evolutionary step for future SMM trials.

In summary, we are attempting to impose discrete ordinal labels on a fundamentally non-quantum disease entity, based on retrospectively analyzed data that is inherently flawed and on arbitrary thresholds, resulting in less-than-precise risk stratification. In active MM, data are well established with clear endpoints, including depth of response, minimal residual disease (MRD) negativity, and biomarker-based progression-free survival; therefore, clinical trial outcomes are easier to compare and interpret. The absence of end-organ damage or symptoms in SMM poses a major challenge for assessing therapeutic benefit. More importantly, and invariably, in early SMM interventional trials, we introduce a lead-time bias, in which a therapeutic benefit may appear without any actual long-term benefit. Any earlier intervention will reset the clock for measuring progression, creating an artifactual benefit of an extended progression-free duration without changing the natural history of the disease, which may not translate into meaningful outcomes, including improvement in overall survival or quality of life, the more clinically relevant endpoints. Therapeutic intervention may very well introduce unnecessary symptoms as adverse effects in an otherwise largely asymptomatic patient. A perfect storm of methodical challenges is being generated, none of which are perhaps truly necessary.

With this critical appraisal of various risk models, let us now examine the key clinical interventional trials in the SMM space, many of which build on these models. We aim to provide a more nuanced understanding of each of these trials, particularly as the fine line between early intervention for benefit and the risk of unnecessary toxicity remains blurred.

## Interventional smoldering myeloma clinical trials

### QuiRedex trial/Spanish PETHEMA trial (NCT00480363)

A randomized phase-III trial published in 2013, represented the first trial to demonstrate progression-free and overall-survival benefits with early intervention in smoldering myeloma (15). Early intervention with lenalidomide-dexamethasone in high-risk SMM significantly reduced progression to symptomatic disease (23% vs 76%) and improved both PFS (HR 0.24 (95% CI 0.14-0.41); mPFS not reached(95% CI 47 months vs NR) vs 23 months (16-31) and OS (HR 0.43, 95% CI 0.21-0.92) at a median follow-up of 75 months, with an acceptable toxicity profile.

The most significant concern centers around the selection criteria, which required the presence of >=95% phenotypically aberrant plasma cells in the bone marrow plasma cell compartment. Given that the trial design predated the revised IMWG definition of multiple myeloma, it raised substantial concerns about whether patients with what is now considered active myeloma (>60% bone marrow plasmacytosis, serum FLC>100, >1 focal lesion on MRI) were inadvertently enrolled. Moreover, the study did not require modern imaging at trial entry (MRI or PET/CT), increasing the likelihood that patients with asymptomatic bone lesions were included in the trial who would otherwise have been treated with standard myeloma therapy. The authors acknowledged this flaw in the trial design, and the independent data monitoring committee’s retrospective review could not entirely rule out the possibility of subtle end-organ bone events that would otherwise have been detected with contemporary imaging techniques. Therefore, these two uncertainties erode confidence in the trial results and may explain the rapid progression rate observed in the non-intervention arm. The combination regimen demonstrated efficacy; however, the specific contribution of lenalidomide to the overall response rate was unclear, given that dexamethasone has demonstrated myeloma activity, a fallacy later addressed by the ECOG trial with its lenalidomide monotherapy approach (14). The protocol amendment limiting treatment duration to 2 years raised further questions about tolerability, although this was not explicitly stated. Moreover, allowing dexamethasone to be added at the time of biochemical progression (observed in the treatment arm) further complicates assessment by introducing treatment heterogeneity within the intervention arm. Of the four total deaths in the treatment group, one treatment-related toxic effect (the respiratory infection) was reported.

Although an overall survival advantage was demonstrated in the intervention arm, multiple potential confounding factors complicated the interpretation of the results. Post-progression treatment was heterogeneous and off-protocol: 81% of patients received bortezomib-based therapy or high-dose therapy with autologous stem cell transplantation at progression, neither of which was uniformly administered or selected, thereby introducing post-progression treatment bias. This raises another point of contention (which was acknowledged by the authors) as patients did not receive a lenalidomide-based combination when they developed ‘active myeloma’ because it was not approved in the newly diagnosed setting at the time. The trial was not powered to assess overall survival as a primary endpoint. The confidence interval was wide (0.21-0.92), indicating uncertainty about the magnitude of the benefit. Quality-of-life metrics were not reported because treatment can introduce symptoms in otherwise asymptomatic patients. Second primary malignancy rates, even though reported to be low (10% in the treatment arm vs 2% in the observation arm), remain concerning and warrant further long-term follow-up. 10 deaths were observed in the treatment arm versus 22 in the observation arm. Of 10 deaths in the treatment arm, 20% (2) were attributed to treatment-related toxicity, one due to a second primary malignancy.

### ECOG ACRIN- E3A06 trial (NCT01169337)

A multi-center, open-label phase III trial demonstrated an improvement in time to progression to symptomatic myeloma with early lenalidomide intervention in patients with ‘High-Risk’ SMM (14). The trial attempted to overcome several limitations of the Spanish PETHEMA trial by evaluating the efficacy of single-agent lenalidomide. Patients with intermediate to HR SMM were randomized 1:1 to receive lenalidomide or observation alone until progression. Lenalidomide monotherapy improved 3-year PFS (91% vs 66%; HR 0.28; 95% CI, 0.12-0.62, p=0.002) and achieved a 50% ORR (95% CI, 39% to 61%), but no OS benefit was demonstrated. Tolerability was poor, with a 40% discontinuation rate and a 45% decline in relative dose intensity.

The trial harbored several methodological flaws that significantly impacted its interpretability and, therefore, its clinical application. Most critically, the eligibility criteria underwent a substantial mid-trial revision after only 21 patients were enrolled, arguably to ‘enhance accrual rate’; the authors admit that the amendment was adopted after the original criteria were found to be too restrictive. The original eligibility requirement necessitated a light chain ratio of under 0.125 or over 8, time period of less than one year since diagnosis, which was then revised to a newer eligibility that required a light chain ratio of <0.26 and or >1.26 time period of less than five years since diagnosis, which represented an expansion of the time-frame by multiple folds and a significant dilution of the biochemical abnormality threshold and therefore implied a diffuse change in the study population mid-stream by introducing a patient population with fundamentally differing characteristics, thereby, introducing conceptual problems. Additionally, the trial did not provide a clear definition of what constituted low-, intermediate-, or high-risk SMM. Patients were enrolled with the initial criteria of having BM plasmacytosis>=10%, abnormal free light chain ratio, and then retrospectively re-assigned them to distinct risk categories using multiple risk stratifying models (Mayo 2008, Mayo 2018, IMWG) representing a circular approach of enrolling a heterogeneous population using a minimal threshold and then recommending treatment *post-hoc (*21–23). The authors acknowledged this flaw by admitting that patients were included in the trial who are no longer considered high risk under the current definition and therefore do not recommend early therapy unless the data are prospectively confirmed.

Furthermore, the tolerability of the treatment arm raises significant concerns about the feasibility of implementing the intervention in real-world clinical practice. A 40% discontinuation rate, an 80% dose reduction, and a 55% relative dose intensity raise questions about whether the intended therapeutic intervention was delivered as intended. The trial, therefore, suggests that lenalidomide monotherapy at its intended dosing in this population is poorly tolerated. There was one treatment-related death in the phase II run-in portion of the trial from pulmonary embolism, which was determined to be therapy-related. Additionally, the absence of a meaningful survival benefit indicates that the trial failed to address its conceptual goal of determining whether early intervention alters the disease trajectory and, therefore, likely introduces lead-time bias in the interventional arm’s progression-free survival benefit.

### CENTAURUS trial (NCT02316106)

The Centaurus trial was a phase II randomized multinational trial designed to evaluate the safety and efficacy of daratumumab as a monotherapy in patients with intermediate to high-risk SMM and laid the foundation for the AQUILA trial (17, 25). One hundred twenty-three patients were randomized 1:1:1 to receive intravenous daratumumab 16mg/Kg on three different dosing schedules (“Extended-intense”, “extended-intermediate”, or “short”). Daratumumab monotherapy failed to meet its primary CR endpoint across all three dosing schedules; the intense, intermediate, and short arms achieved ORRs of 56%, 53%, and 38%, 24-month PFS rates of 90%, 82%, and 75%, and grade 3–4 adverse events in 44%, 27%, and 15% of patients respectively, with only 4.9% overall discontinuing due to toxicity. There were two deaths total across the three arms; one each in the intermediate and short arms, both of which were attributed to disease progression.

The major problem with the trial was the study’s failure to meet its co-primary endpoint of CR >15% (the threshold for the null hypothesis). Even with extended follow-up, the responses did not deepen sufficiently to achieve CR ranging from 0%-9.8%, which clearly indicates that daratumumab monotherapy failed to eradicate MM in its precursor state, which was the initial assumption, considering the ambitious alternate hypothesis CR target rate of >35%. The authors explicitly acknowledged that “single-agent daratumumab may not be sufficient to eradicate SMM” yet proceeded to state that daratumumab sufficiently achieved disease control and therefore was successful, which creates a philosophical conundrum where an initial assumption and goal of eradicating disease and disease control was subsequently mis-translated into “failing to achieve disease eradication and yet succeeded to achieve disease control”. Further, the risk stratification utilized by CENTAURUS suffers from similar drawbacks to AQUILA; there was no clear distinction between intermediate- and high-risk SMM. A mid-study protocol amendment that adds FLC > 100 mg/L as an eligibility criterion further complicates the interpretation of the trial results by altering the study population during enrollment. For faster accrual, the study authors knowingly included patients with an ‘intermediate’ risk of progression who may have a more indolent disease biology, thereby subjecting this sub-population to unnecessary treatment and creating disease heterogeneity that dilutes treatment effects. The absence of a control arm, while acceptable for a phase II study, limits its ability to demonstrate treatment effect and restricts the interpretation of the results to hypothesis-generating rather than proving efficacy. At the time of screening, 43.2% failed to meet eligibility criteria because they had active MM. This high rate of active disease, initially thought to have SMM, raises profound concerns regarding disease classification in routine practice. Such misclassification attests to the increasingly blurred line between high-risk SMM and active MM following the 2014 revision of the IMWG criteria defining active multiple myeloma. The dichotomous distinction based on biomarkers erodes confidence in our ability to distinguish a coherent disease that needs treatment from a disease entity that is nothing more than an artificial construct.

The pharmacokinetic data reported by the trial is incredibly valuable, as it informs us of the impact of treatment intensity on disease control. One may deduce that once the therapeutic level of daratumumab diminishes, disease control is lost, indicating that daratumumab, for the most part, achieves disease control rather than eradication. This implies that to gain and maintain benefit, continuous treatment with daratumumab is necessary; otherwise, the benefit is lost. Such a hypothesis may also be applicable to the active MM space, where maintenance daratumumab is increasingly used post-stem cell transplantation.

### AQUILA trial (NCT03301220)

The Aquila trial represents the most contemporary and methodologically sophisticated clinical trial conducted to date (25). SMM risk stratification was precise and well defined. 390 patients were randomized 1:1 to receive either subcutaneous daratumumab monotherapy or active monitoring alone.

Subcutaneous daratumumab demonstrated a 51% reduction in risk of progression or death (HR 0.49, 95% CI, 0.36-0.67, p<0.001), with superior 5-year PFS (63% vs 41%) and OS (93% vs 87%; HR 0.52, 95% CI, 0.27-0.98), over observation, an ORR of 58.5%, and grade 3–4 adverse events in 40% vs 30% of patients, respectively.

Even though the Aquila trial was well-conceived and executed, it has several limitations. The most fundamental concern centers around the risk stratification criteria deployment, which suffers from the same binary dichotomy that plagues other SMM trials, but the inclusion of IgA myeloma smoldering myeloma as a stand-alone, high-risk criterion in addition to bone marrow plasmacytosis of >=10% represents an over-simplification of the understanding that IgA myeloma tends to be associated with a high-risk disease biology. Patients with IgA SMM with a low disease burden may inappropriately be classified as high-risk, leading to over-treatment. The progression-free survival endpoint, while statistically significant, shared the same disadvantage as other interventional SMM trials: lead-time bias. The OS benefit, while statistically significant and encouraging, needs cautious interpretation given the small total number of deaths (15 versus 26) and a wide confidence interval (0.27-0.98); moreover, the trial was not powered for OS. The survival benefit could also be confounded, as post-progression access to subsequent therapies may be limited, given that this was a multinational study and that daratumumab may not be available as standard therapy in many of the countries where the trial was conducted. Of the 105 patients in the control arm who subsequently received treatment, only 18% received a daratumumab-containing regimen. In the daratumumab group, 2 patients (1.0%) died from adverse events, both from COVID-19-related causes. In the active-monitoring group, 4 patients (2.0%) died from adverse events: pulmonary edema, cardiac arrest, pulmonary embolism, and cardiac failure.

Comparison between CENTAURUS, AQUILA, and other more intensive interventional approaches in SMM raises further philosophical questions. The contrast in ORR between single-agent daratumumab and other intensive combination therapies is striking. While single-agent daratumumab produced an ORR of 50-60%, combination therapies in high-risk SMM achieved an ORR of 97%, with substantial deepening to MRD negativity, which may therefore be preferable.

### Other single-arm early-phase studies

Several early-phase studies have evaluated intensive combination approaches in high-risk SMM. The ASCENT trial (daratumumab, carfilzomib, lenalidomide, and dexamethasone; n=87) achieved a 97% ORR and a 3-year PFS of 90%, but only 47% of patients completed the intended 2-year course, 51% experienced non-hematologic toxicities, and the absence of a control arm limits interpretability (26). The ISAMR trial (isatuximab-lenalidomide, n=61) demonstrated an ORR of 89% with a manageable toxicity profile (27), while the CAR-PRISM safety run-in reported 100% ORR and universal MRD negativity at 10^-6^ with cilta-cel in six patients (28), and GEM-CESAR and NIH-KRD demonstrated deep MRD-negative responses using KRd-based intensive approaches with or without autologous transplant (8, 16). However, all are single-arm phase II studies without control arms, with enrollment heterogeneity due to inconsistent risk stratification, and several included patients who would now meet criteria for active myeloma under SLiM-CRAB, limiting the ability to draw definitive conclusions. ITHACA, a randomized phase III trial of isatuximab, lenalidomide and dexamethasone versus lenalidomide and dexamethasone, represents another methodologically rigorous ongoing study in this space, though results remain immature (29). Taken together, these trials generate important hypotheses but cannot yet inform practice.

Details regarding the above trials are summarized in **Table 1**.

**Table 1:** Smoldering multiple myloma (SMM) trials.

Study	Design	SMM risk	Definition of progression	Intervention	Primary endpoint	Population size	Efficacy data	Median follow up
Lenalidomide singlet and doublet regimens								
ECOG Acrin E3A06 (15)	Phase 2/3, randomized, open-label multicenter study	Intermediate- or high-risk Risk defined as: BM plasmacytosis ≥10% and abnormal serum FLC ratio of <0.26 or >1.65	Presence of biochemical disease progression as defined by IMWG criteria (CRAB)^a^	Lenalidomide 25 mg PO QD x days 1-21 (28-cycles)^b^ vs Observation Continued until disease progression, toxicity, or any-cause withdrawal	PFS	N=182 (treatment arm: n=90; observation: n=92) Phase 2 safety run-in: N=44	ORR - 50% PFS - 91% vs 66% in the control group at 3 years	35 months
QUIREDEX (14)	Phase 3, randomized, open-label multicenter study	High-risk Risk defined as: BM plasmacytosis ≥10% and a monoclonal component^c^ or only 1 of these 2 criteria + ≥95% phenotypically aberrant BMPC with reductions in 1-2 uninvolved immunoglobulins >25% vs normal values	Presence of symptoms Asymptomatic biologic progression, defined as an increase of >25% in the monoclonal component vs lowest recorded value during treatment without the presence of symptoms	Induction (Lenalidomide 25 mg PO QD x days 1-21 + dexamethasone 20 mg PO QD x days 1-4, 12-15 for nine, 28-day cycles) and Maintenance (lenalidomide 10 mg PO QD x days 1-21) for 2 years) Vs Observation Continued until disease progression, toxicity, or any-cause withdrawal	Time to progression to symptomatic disease	N=119 (treatment arm: n=57; observation: n=62)	ORR - 20% PFS - not reached in treatment arm vs 23 months in intervention arm	40 months
Anti-CD38-based therapy								
AQUILA (25)	Phase 3, randomized, open-label, multicenter study	High-risk Risk defined as: BM plasmacytosis ≥10% and presence of at least one of the following: serum M-protein level ≥30 g/L; IgA smoldering MM, immunoparesis with reduced levels of 2 uninvolved immunoglobulin isotypes; serum FLC ratio of 8-<100; or clonal BMPC of >50% to <60%	Assessed by independent review committee in accordance with IMWG SLiM-CRAB diagnostic criteria^a^	Daratumumab 1800 mg SC (with recombinant human hyaluronidase) weekly x Cycles 1-2, every other week in Cycles 3-6, every 4 weeks thereafter for a total of 39 cycles (36 months) Vs Active monitoring Continued until disease progression	PFS	N=390 (treatment arm: n=194; active monitoring: n=196)	ORR - 58.5% in the treatment arm PFS - 63.1% vs 40.8% at 5 years OS- 93% vs 86.9% at 5 years	65.2 months
CENTAURUS (17)	Phase 2, randomized, open-label, multicenter study	Intermediate- or high-risk Risk defined as: BM plasmacytosis ≥10% to <60% and at least 1 of the following: serum M-protein level ≥3 g/dL (IgA ≥2 g/dL); urine M-protein >500 mg/24 hours; abnormal FLC ratio (ie, 0.02 to 0.126 or 8 to <100) with serum M-protein 1-<3 g/dL; or absolute involved serum FLC ≥100 mg/L with abnormal FLC ratio	Defined per 2014 IMWG criteria (SLiM-CRAB)^a^ + additional IMWG FLC progression criteria (2 consecutive visits of involved / uninvolved serum FLC of ≥100 mg/L, ≥25% increase from nadir between involved and uninvolved FLC levels, with absolute increase of >10 mg/dL)	**Intense** ( daratumumab 16 mg/kg qw x Cycle 1, q2w x Cycles 2-3, q4w x Cycles 4-7, q8w x Cycles 8-20) Vs **Intermediate** (daratumumab 16 mg/kg qw x Cycle 1, q8w x Cycles 2-20) Vs **Short** ( daratumumab 16 mg/kg qw x Cycle 1 only)	Co-primary endpoints: CR rate and PD/death rate	N=123 (Intense arm: n=41; Intermediate arm: n=41; Short arm: n=41)	ORR - 56.1%, 53.1% and 37.5% in the three arms PFS- 89.9%, 82% and 75.3% in the three arms at 24 months	15.8 months (August 8, 2017 cutoff) 25.9 months (June 29, 2018 cutoff)
ISAMAR (27)	Phase 2, two-staged, single-arm, non-randomized, multicenter study	High-risk Risk defined as: Immunoparesis and ≥95% abnormal BMPC	Not defined in abstract	Stage 1: isatuximab 20 mg/kg IV x days 1,8,15,22 x Cycle 1, days 1,15 x Cycles 2-6, day 1 x Cycles 7-30 Stage 2: Stage 1 isatuximab regimen + lenalidomide 25 mg PO QD x days 1-21 for Cycles 1-6	ORR	N=61 (stage 1: n=25; stage 2: n=36)	ORR- 89% with stage II PFS - 49.3 months with stage II	Stage 1: 49 months Stage 2: Not reported in abstract
Fixed-duration dara-KRd regimen								
ASCENT (26)	Phase 2, single-arm, nonrandomized, multicenter study	High-risk Risk defined as: meeting IMWG 20/2/20 staging^d^ or ≥9 on IMWG scoring system^e^	Not defined in abstract	Induction (cycles 1-6)^f^: Carfilzomib 56 mg/m2 IV x days 1, 8, 15 + Lenalidomide 25 mg PO QD x days 1-21 + daratumumab 16 mg/kg IV QW for cycles 1-2 and QOW for cycles 3-6 + dexamethasone 40 mg QW Consolidation (cycles 7-12): Carfilzomib 56 mg/m2 IV x days 1, 8, 15 + Lenalidomide 25 mg PO QD x days 1-21 + dexamethasone 20 mg QW Maintenance (cycles 13-24): Lenalidomide 10 mg PO QD x days 1-21 + daratumumab 16 mg/kg IV once per cycle	sCR rate	N=87	ORR - 97% with 37% sCR, 26% CR and 29% VGPR PFS - 89.9% at 3 years	25.8 months
Transplant-inclusive regimen								
NCT01572480 (16)	Phase 2, single-arm, non-randomized, single-center, study	High-risk Risk defined based on Mayo Clinic, Spanish, and/or Rajkumar, Landgren, and Mateos criteria^g,h,i^	Defined per 2014 IMWG criteria (SLiM-CRAB)^a^	Eight 28-day cycles of KRd: Carfilzomib 36 mg/m^2^ (first 2 doses: 20 mg/m^2^) IV on days 1, 2, 8, 9, 15, 16) + Lenalidomide 25 mg PO QD x days 1-21 + Dexamethasone (oral or IV) 20 mg biw x cycles 1-4; 10 mg biw x cycles 5-8 SCT after 4 cycles for eligible patients Maintenance: lenalidomide 10 mg PO QD x days 1-21 for 24, 28-day cycles Indefinite observation period after maintenance therapy	MRD-negative CR rate	N=54	ORR - 100% with 70.4% CR PFS - 5.5 years and the 8-year probability of being free from progression was 91.2%	31.9 months
GEM-CESAR (8)	Phase 2, single-arm, open-label, non-randomized, multicenter study	High-risk Risk defined as: Mayo 2008 or GEM-Pethema criteria^g,h^	Biochemical progression defined as biochemical relapse or progressive disease according to IMWG criteria,^a^ or ultrasensitive MRD relapse, defined as MRD reappearance ≥2 months apart	Induction (KRD; 6, 28-day cycles): carfilzomib 20/36 mg/m2 IV QD x days 1,2,8,9,15,16 + lenalidomide 25 mg PO QD x days 1-21 + dexamethasone 40 mg QW PBSC mobilization with G-CSF planned after 4th induction cycle Melphalan 200 mg/m2 conditioning, followed by ASCT Consolidation: KRd (same as induction; 2 cycles) initiated 3 months after ASCT Maintenance: lenalidomide 10 mg PO QD x days 1-21 + dexamethasone 20 mg QW	uMRD rate after ASCT	N=90	ORR - 94% with 64% CR at the end of maintenance PFS- 94% at 70 months; 40% experienced biochemical progression OS - 92% at 70 months	70.1 months
BCMA-directed CAR T-cell therapy								
CAR-PRISM (28)	Phase 2, single-arm, non-randomized, single-center study	High-risk Risk defined as: meeting IMWG 20/2/20 criteria or ≥9 on IMWG scoring system, high-risk FISH, evolving pattern; or GEM-PETHEMA criteria^d,e,g^	Not defined in abstract	First 3 patients: lymphodepletion (fludarabine + cyclophosphamide) then cilta-cel 0.5 x 10^6^ CAR-positive T-cells/kg Next 3 patients: lymphodepletion (fludarabine + cyclophosphamide) then cilta-cel 0.75 x 10^6^ CAR-positive T-cells/kg	Safety	N=6 (dividing into two, 3-patient cohorts)	ORR- 100% with CR of 50%	6 months

Bold text indicates therapy-class groupings.

BIW, twice weekly; BM, bone marrow; BMPC, bone marrow plasma cell; CAR, chimeric antigen receptor; CR, complete response; CT, computed tomography; FISH, fluorescence in situ hybridization; FLC, free light chain; LLN, lower limit of normal; PD, progressive disease; PET, positron emission tomography; PFS=progression-free survival; PO, by mouth; QD, once daily; QOW, once every other week; QW, once weekly; ULN, upper limit of normal; uMRD, undetectable measurable residual disease.

^a^IMWG 2014 (SLiM-CRAB) criteria define MM as clonal BMPC ≥10% or biopsy-proven bony or extramedullary plasmacytoma and ≥1 of the following myeloma-defining events: evidence of end-organ damage (hypercalcemia [>1 mg/dL higher than ULN or >11 mg/dL]; renal insufficiency [creatinine clearance <40 mL/min or serum creatinine >2 mg/dL]; anemia [HgB >20 g/L below LLN or HgB <100 g/L]; presence of one or more osteolytic lesions on skeletal radiography/CT/PET-CT) or malignancy markers (clonal BMPC ≥60%; involved:uninvolved serum FLC ratio ≥100; ≥1 focal lesions on MRI studies (10).

^b^Investigators encouraged patients to undergo stem cell mobilization after 4-6 cycles of therapy.

^c^Monoclonal component, defined as IgG level ≥3 g/dL, IgA level ≥2 g/dL, or urinary Bence Jones protein level of >1/24 hours.

^d^Mayo 20/2/20 staging is based on the following criteria to predict the risk of progression to active MM: serum M-protein >2 g/dL, involved to uninvolved free light-chain ratio >20, and marrow plasma cell infiltration >20% (22).

^e^The IMWG scoring system assigns scores based on key lab values, which are as follows: FLC: 0-10 (0 points), >10-25 (2 points), >25-40 (3 points), >40 (5 points); M-protein 0-1.5 (0 points), >1.5-3 (3 points), >3 (4 points); BMPC: 0-15 (0 points), >15-20 (2 points), >20-30 (3 points), >30-40 (5 points), >40 (6 points); FISH abnormality (2 points) (23).

^f^Daratumumab also given once per cycle during induction.

^g^GEM-PETHEMA criteria include scoring based on the proportion of bone marrow plasma cells with abnormal cell surface markers (<95%, zero points; ≥95%, one point) and the presence (one point) or absence (zero points) of immunoparesis (20).

^h^Mayo 2008 criteria include scoring based on BMPC percentage ≥10% (one point), serum M-protein ≥3 g/dL (one point), and serum immunoglobulin FLC ratio either <0.125 or >8 (one point) (21).

^i^Rajkumar, Landgren, and Mateos criteria define high-risk SMM as the presence of clonal BMPC ≥10% and ≥1 of the following: serum M protein ≥30g/L; IgA SMM; immunoparesis with reduction of 2 uninvolved immunoglobulin isotypes; serum involved/uninvolved FLC ratio ≥8 (but <100); progressive increase in M protein level (evolving type of SMM; increase in serum M protein by ≥25% on 2 successive evaluations within a 6-month period); clonal BMPCs 50%-60%; abnormal PC immunophenotype (≥95% of BMPCs are clonal) and reduction of ≥1 uninvolved immunoglobulin isotypes; t(4;14) or del(17p) or 1q gain; increased circulating PCs; MRI with diffuse abnormalities or 1 focal lesion; PET-CT with focal lesion with increased uptake without underlying osteolytic bone destruction.

## Discussion

All trials create confusion about clinical benefit. Should biochemical progression, as defined by the IMWG for multiple myeloma, need therapeutic change or escalation for patients with SMM? What constitutes biochemical progression as defined by the IMWG for multiple myeloma may not necessarily apply to smoldering myeloma when it comes to changes in therapy aimed at achieving meaningful clinical benefit. Clinical investigators for some of the SMM trials recommend incorporating the IMWG definition of biochemical progression into the SMM space, but this notion sets a dangerous precedent, as it is unclear whether biochemical progression in such a state predicts end-organ events through meaningful acceleration to symptomatic disease. Such an approach provides an excellent example of Munchausen’s Trilemma, in which treatment that prevents biochemical progression appears successful regardless of meaningful clinical outcomes. Furthermore, the use of MRD negativity as a benchmark for predicting outcomes in smoldering myeloma is not well defined. In active myeloma, it is a vital biomarker that has been repeatedly validated as a clinically significant endpoint predicting PFS risk; however, this relationship may not hold in SMM, where disease biology and evolution may differ.

Furthermore, as emerging technologies may allow us to distinguish active myeloma that has not yet met CRAB criteria from truly indolent disease, the methodology for analyzing accumulating data from interventional smoldering trials will need to change accordingly. Pioneering work by Kumar et al. and Ghobrial et al. (genomic profiling and 3D telomere profiling) and the incorporation of these markers into outcome prediction are promising approaches that may eventually change our perception of smoldering myeloma (30, 31).

SMM being more of a disease continuum than a single disease entity by itself may perhaps be better defined by a dynamic risk assessment that would consider established trends over time, incorporation of the rate of change of parameters, addition of continuous risk scores rather than binary values, consideration for parameters weighed by their prognostic significance (as evaluated by the IMWG risk stratification of SMM). While operationally convenient, using a binary threshold leads to inappropriate risk stratification and incorrect therapeutic decisions. Perhaps this conceptual limitation may have produced a potential artifactual progression and survival benefit in a subgroup of patients with an inherently indolent disease biology who received therapy. Furthermore, several FISH and NGS changes are known to be associated with an increased risk of progression to active disease, neither of which was considered in any of the trials (30, 32–35). As our understanding of the disease evolves, a more nuanced approach to risk stratification is needed to accurately identify patients who would benefit from earlier intervention. The therapeutic sequence in the SMM trials creates a unique consequence for a newly diagnosed disease with either single-agent or multi-agent refractoriness. The modern quadruplets, which are now widely used worldwide and considered the new standard of care, are therefore rendered less viable. Anti-CD38-based combination therapies have shown unprecedented response rates and improved survival following treatment initiation. Historically, the use of single-agent therapies in multiple myeloma has largely been discouraged, given poor efficacy data and survival outcomes (36, 37). The choice of single-agent lenalidomide or daratumumab for the treatment of SMM therefore represents a significant deviation from this widely held philosophy, particularly given the low overall response rate reported in earlier studies (38). Combination therapies have consistently demonstrated superiority for multiple myeloma (39–41). Given that myeloma is a clonally heterogeneous disease, even at precursor stages, a multi-targeted approach is preferable to reduce the risk of resistance development. Additionally, synergistic effects with combination therapies are well proven in multiple myeloma. We recognize that some of these questions are being addressed in several ongoing studies (ASCENT, ITHACA, PRISM, and DETER-MM), with all these trials demonstrating superior response rates to single-agent therapies (26, 28). Moreover, the financial implications of treating high-risk SMM even with limited-duration therapy require serious consideration, given that a substantial portion of the untreated patients are progression-free at prolonged follow-up as observed in several of these trials. A predominant portion of healthcare utilization is therefore being invested without benefit, which would otherwise be directed towards other aspects of myeloma care.

All SMM trials also present several practical challenges regarding future treatment access. One such challenge is insurance coverage for subsequent therapies following progression. Since these patients never met the criteria for active disease, insurance coverage at progression may become problematic. This issue also extends to trial eligibility – patients treated in the SMM state may be excluded from future trials designed for active MM. Lastly, given the rapidly evolving treatment landscape in MM, early intervention may prematurely lock patients into current therapeutic paradigms. Patients may therefore benefit more from waiting until progression, when newer and potentially more effective therapies may become available, particularly given that the survival trend for patients with myeloma has improved significantly over time (42–45,46).

In November 2025, the Food and Drug Administration (FDA) approved subcutaneous daratumumab monotherapy for high-risk smoldering multiple myeloma, based on the results of the AQUILA trial, marking the first approved therapy for this indication. While this is an important milestone, several concerns remain regarding this approval, particularly regarding community practice. The commercial availability of an FDA-approved agent will exert pressure on clinicians and patients alike, normalizing the treatment of smoldering myeloma, whose natural history is inconsistent and poorly understood.

None of the four randomized trials meaningfully addresses age-appropriate patient selection or comorbidity burden. ECOG E3A06 is the only one to even report an age subgroup, and that analysis was underpowered. The eligibility criteria across all trials required ECOG PS 0–1 (or 0–2 in QuiRedex), which systematically excluded frail or heavily comorbid patients, indicating that the trial populations are inherently younger and fitter than the real-world SMM population.

## Conclusions

In conclusion, while the SMM trials represent critical steps in the evolution of our understanding of early intervention in SMM, it raises several key issues including the problematic risk assessments, potential over treatment of an indolent disease particularly in an era when we are seeing unprecedented responses for patients with newly diagnosed myeloma and creating a unique situation of single or multi-agent refractoriness in a newly diagnosed disease, all of which warrant careful re-assessment. Across all four randomized trials, statistically significant improvements in PFS were observed, and two trials reported OS benefit (QuiReDex and Aquila); however, we should interpret these results with caution due to methodological limitations. Inconsistent and outdated risk stratification models were used to enroll patients with a heterogeneous disease biology and, in some instances, patients who would now meet the criteria for active myeloma under the newer IMWG definition. Lead-time bias is a universal confounder of the PFS endpoint, and none of the trials were adequately powered to demonstrate an OS advantage. Few interventional trials address key questions regarding quality-of-life assessment, which is an important consideration when treating patients with smoldering myeloma. A more nuanced approach to risk stratification is necessary, particularly if we are considering therapeutic intervention for SMM to avoid unnecessary biological and financial toxicities in patients with an indolent disease. If we appropriately identify the actual high-risk SMM in a timely fashion, use of combination therapies (as we do with newly diagnosed multiple myeloma) would undoubtedly be more favorable than a single agent. Practical challenges, including insurance coverage at progression and future trial eligibility in the active myeloma space, need to be considered. Prematurely capturing patients with contemporary therapies in SMM may compromise future options at progression, which may prove more effective. Until then, the age-old principle of “Primum non nocere”- “first, do no harm” should guide us in balancing the risks of early intervention against the benefits.

In our practice, we favor close monitoring and observation over therapeutic intervention for patients with high-risk smoldering myeloma, consistent with the principle of primum non nocere outlined throughout this review. In the rare circumstance where serial assessments demonstrate a sustained and progressive rise in paraprotein burden with a trajectory strongly suggestive of imminent end-organ compromise, an individualized decision to initiate therapy may be considered, though this remains the exception rather than the rule, and is guided by clinical judgment rather than risk model classification alone.
